# Role of Tau Protein in Remodeling of Circadian Neuronal Circuits and Sleep

**DOI:** 10.3389/fnagi.2019.00320

**Published:** 2019-11-21

**Authors:** Mercedes Arnes, Maria E. Alaniz, Caline S. Karam, Joshua D. Cho, Gonzalo Lopez, Jonathan A. Javitch, Ismael Santa-Maria

**Affiliations:** ^1^Taub Institute for Research on Alzheimer’s Disease and the Aging Brain, Columbia University, New York, NY, United States; ^2^Department of Pathology and Cell Biology, Columbia University, New York, NY, United States; ^3^Department of Psychiatry, Vagelos College of Physicians and Surgeons, Columbia University, New York, NY, United States; ^4^Department of Genetics and Genomic Sciences, Icahn School of Medicine at Mount Sinai, New York, NY, United States; ^5^Division of Molecular Therapeutics, New York State Psychiatric Institute, New York, NY, United States; ^6^Department of Pharmacology, Vagelos College of Physicians and Surgeons, Columbia University, New York, NY, United States

**Keywords:** tau, circadian rhythm, sleep, PDF, structural plasticity

## Abstract

Multiple neurological, physiological, and behavioral functions are synchronized by circadian clocks into daily rhythms. Neurodegenerative diseases such as Alzheimer’s disease and related tauopathies are associated with a decay of circadian rhythms, disruption of sleep patterns, and impaired cognitive function but the mechanisms underlying these alterations are still unclear. Traditional approaches in neurodegeneration research have focused on understanding how pathology impinges on circadian function. Since in Alzheimer’s disease and related tauopathies tau proteostasis is compromised, here we sought to understand the role of tau protein in neuronal circadian biology and related behavior. Considering molecular mechanisms underlying circadian rhythms are conserved from *Drosophila* to humans, here we took advantage of a recently developed tau-deficient *Drosophila* line to show that loss of tau promotes dysregulation of daily circadian rhythms and sleep patterns. Strikingly, tau deficiency dysregulates the structural plasticity of the small ventral lateral circadian pacemaker neurons by disrupting the temporal cytoskeletal remodeling of its dorsal axonal projections and by inducing a slight increase in the cytoplasmic accumulation of core clock proteins. Taken together, these results suggest that loss of tau function participates in the regulation of circadian rhythms by modulating the correct operation and connectivity of core circadian networks and related behavior.

## Introduction

Circadian rhythms impose daily cycles to many behaviors and physiological processes in a wide variety of organisms. In mammals, the master biological clock is a group of about 20000 neurons that form a structure called the suprachiasmatic nucleus (SCN). In *Drosophila melanogaster* (fruit fly) the small ventral Lateral Neurons (sLNvs) are the master circadian pacemaker cells that set the pace of locomotor activity rhythms (Stoleru et al., 2005). At the molecular level, the molecules that regulate daily circadian behavioral rhythms are well known and conserved between mammals and insects like *Drosophila*. These molecular clocks consist of interlocked transcriptional-translational feedback loops that drive circadian rhythms in gene expression (Hardin, 2011).

The dysregulation of circadian clocks contributes to the decline in optimal functioning of the nervous system, ultimately leading to a reduction in the quality of life due to cognitive impairments and emotional stress (Touitou and Haus, 2000; Hastings et al., 2003; Hafycz and Naidoo, 2019). Therefore, since damaged circadian rhythms and sleep/wake cycles have been associated with an increase in disease susceptibility (Hastings et al., 2003; Gibson et al., 2009) it is important to determine the rather complex source of age-related circadian or sleep/wake dysfunction. Some of the observed sources of circadian dysfunction are low-functioning neuronal connections or loss of neurotransmitter or neuropeptide production, transport, or secretion (Kolker et al., 2003).

Maintenance of circadian rhythms requires exquisite synaptic coordination at multiple molecular and cellular levels of brain organization. The emergence of this complex circadian clock network requires both stable and dynamic properties of the neuronal cytoskeleton (Shweiki, 1999). Microtubules are key components of the cytoskeleton and their localization, mass, and dynamics are very important in many intracellular processes in neuronal health and disease (Brandt and Bakota, 2017; Dent, 2017). In addition, it has been demonstrated that microtubules have an influence on circadian activity patterns (Jarzynka et al., 2006, 2009). Microtubule stability is regulated by various microtubule-associated proteins (MAPs), like tau, MAP1B and MAP2 (Matus, 1988). Among them, tau protein adds stability and dynamic properties to the neuronal cytoskeleton, facilitating the maturation and establishment of synaptic networks involved in a large number of complex behaviors (Voelzmann et al., 2016; Tracy and Gan, 2018).

The multiple functions and different locations of tau in neurons have revealed novel insights into its importance in a diverse range of molecular pathways including cell signaling, synaptic plasticity, and regulation of genomic stability (Guo et al., 2017). However, little is currently known about the role tau protein plays in circadian regulation and sleep and the possible mechanisms involved (Cantero et al., 2010). Conversely, only few reports have shown how sleep regulates tau (Holth et al., 2019).

*Drosophila* has been used as a powerful model system to investigate *in vivo* the role of proteins linked to human diseases, including AD (Gistelinck et al., 2012; Rincon-Limas et al., 2012). Interestingly, the human tau protein has a fly homolog called *Drosophila* tau (dTau) which also displays microtubule-binding properties (Heidary and Fortini, 2001). Moreover, to further dissect the functions of the endogenous dTau protein, a new tau knock-out (Tau^–/–^) fly line has been generated by homologous recombination (Burnouf et al., 2016).

The work presented here is focused on addressing the current gap in our knowledge on the role tau protein plays in regulating circadian rhythms and sleep patterns in *Drosophila*. Using this new *Drosophila* tau KO (dTau^–/–^) line, we found alterations in daily circadian activities and dysregulation of sleep accompanied with molecular and structural changes in circadian pacemaker neurons, suggesting a new role for tau protein in circadian regulation and sleep. Taken together, our results demonstrate that tau in *Drosophila* has an impact on behavioral rhythms and sleep patterns, likely due to its role in modulating the structural plasticity of the terminal projections of circadian pacemaker neurons, demonstrated by the temporal dynamics of dTau levels in sLNv neurons.

## Materials and Methods

### *Drosophila* Stocks

All *Drosophila* stocks were maintained on standard food (Bloomington recipe, Archon Scientific) in incubators at constant 70% relative humidity and 25°C on a 12-h/12-h light/dark cycle (unless otherwise specified). *Drosophila* dTau knockout line (dTau^–/–^) was generated and kindly provided to us by Prof. Dr. Linda Partridge (Burnouf et al., 2016). dTau^–/–^ line was isogenized and backcrossed for more than 10 generations with control line w^1118^ (Stock #5905) obtained from the Bloomington Drosophila Stock Center (Indiana University, United States). dTau-GFP line (Stock #60199) was also obtained from Bloomington Drosophila Stock Center.

### Measurement of *Drosophila* Circadian Activity

**FIGURE 1:**
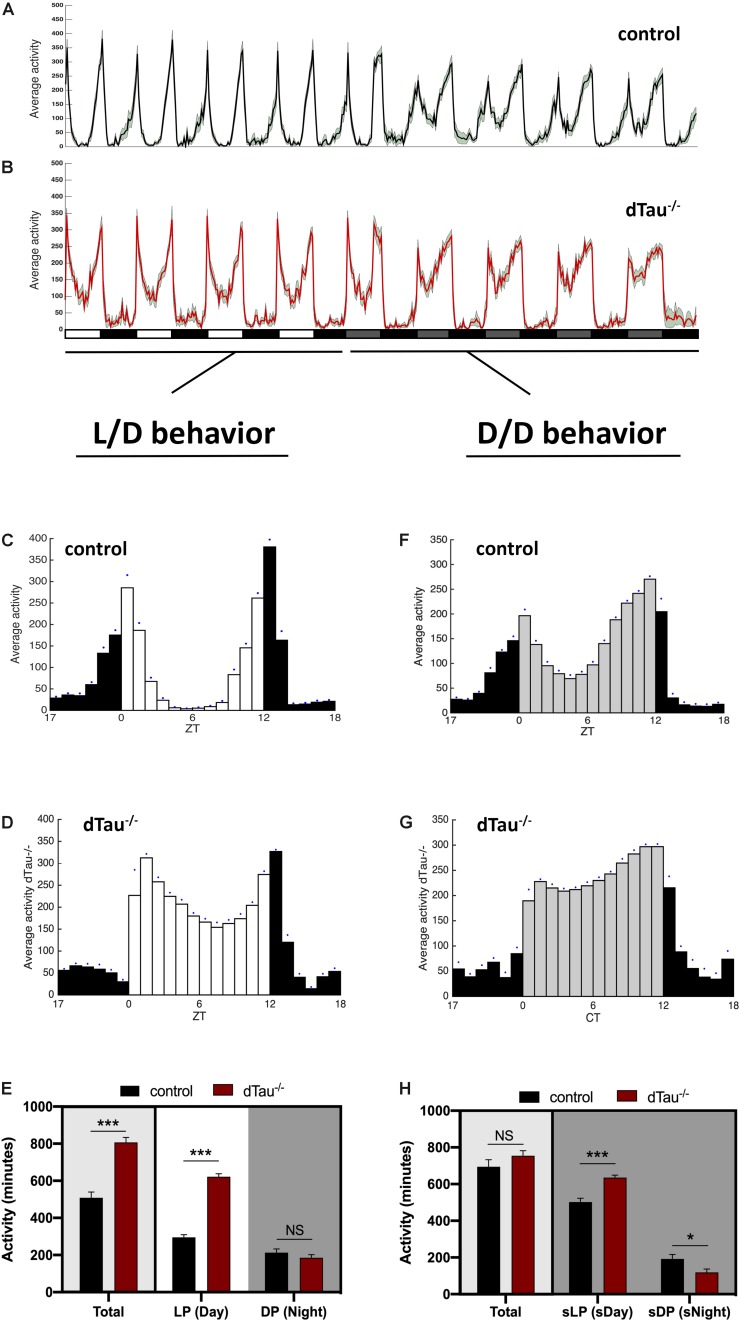
Effect of tau deficiency on circadian activity in *Drosophila*. **(A,B)** Averaged activity profiles across four 12 h LD cycles followed by another four DD cycles, of 7 day-old male flies from the denoted genotypes (w1118 in black and dTau^–/–^ in red). White rectangles represent light period (LP), or day, and black rectangles represent dark period (DP) or night. During DD cycles, subjective light period (sLP) is represented in gray, and subjective dark period (sDP) in black. Activity peaks in dTau^–/–^ flies show a different pattern when compared to controls, with noticeably more activity values during the “siesta” period in the light period during LD cycles, and the subjective day during the DD cycles. **(C–H)** Averaged activity profiles across five LD and DD cycles of the corresponding genotypes. White bars represent the light period (day), and black bars represent the dark period (night) in LD cycles **(C,D)**. During DD cycles **(F,G)** subjective daytime is represented in gray. Blue dots represent SEM for each Zeitgeber time. Both in LD and DD cycles, dTau^–/–^ flies **(D,G)** were more active than controls **(C,F)** during the day and subjective daytime, with higher activity values and shows affection of the normal “siesta” pattern that control flies elicit during this particular period. **(E,H)** Total averaged locomotion activity quantification demonstrates that dTau mutant flies were significantly more active than controls, specifically during the LP in LD cycles. In DD cycles, dTau mutant flies were also significantly more active than controls during both the sLP and the sDP. However, no changes were found in total locomotor activity during DD cycles. Data represent mean and SEM analyzed with non-parametric Mann–Whitney statistical test, with ^∗^*p* < 0.05 and ^∗∗∗^*p* < 0.001 or NS if no statistical significance (*n* = 21–27 flies).

Circadian activity of flies was measured as previously described (Chiu et al., 2010). Briefly, single 7 days-old male flies were placed in 5 × 65 mm glass tubes that fit a custom-built Multibeam Activity Monitors (DAM5M, Trikinetics Inc.) with four sets of infrared beams for activity detection. All tubes contained 2% agarose with 5% sucrose food. The monitors were connected to a computer to record beam breaks every minute for each animal using standard data acquisition software (DAMSystem 3, Trikinetics Inc.). Beam breaks occur due to locomotor activity of the single flies through the tubes. At the conclusion of the experiment, raw binary data collected was processed using DAM FileScan 111X (Trikinetics Inc.) and summed in 30 min bins when analyzing circadian parameters. DAM5M monitors were housed in a 25°C and 70% relative humidity incubator. Day/night activity was measured by maintaining the flies in a 12 h Light/Dark (LD) cycle for 5 days. Circadian activity rhythms was measured under constant darkness (DD) for 6–9 days after an entraining period of 5 days in LD cycles. Data analysis of Drosophila activity shown in actograms and eduction graphs (Figure 1) were performed using FaasX software. Further analyses of circadian activity in DD conditions (Table 1) were done in Matlab using the SCAMP scripts developed by Vecsey lab from Skidmore College (Donelson et al., 2012).

**TABLE 1:** Circadian behavior analysis of flies in D/D conditions.

**Genotype**	**Period (h)**	**RI**	**RS**	**% rhythmic**	**n**
control	23.99 ± 0.03	0.5593 ± 0.03	5.264 ± 0.37	100%	22
dTau^–/–^	24.04 ± 0.09	0.5816 ± 0.03	5.437 ± 0.42	94.4%	25

*Data are mean ± SEM. Table representing period length, the rhythmic index (RI), the rhythmic strength (RS), the percentage of rhythmic flies, and the number of total flies used for the study.*

### Sleep Analysis

For sleep analysis, 7 day-old male Drosophila locomotor activity data was collected and summed in 1 min bins. Sleep was quantified in Matlab using SCAMP scripts (Donelson et al., 2012). Wakefulness was defined as any period of at least 1 min characterized by activity (≥1 count/min) and sleep was defined as any period of inactivity (0 counts/min) lasting ≥5 min (Hendricks et al., 2000; Shaw et al., 2000; Van Swinderen et al., 2004). Sleep parameters analyzed were total sleep, number of sleep episodes, mean sleep episode duration (is a measure of how consolidated the sleep is and can illustrate the quality of sleep), total time awake and sleep latency (time in minutes from lights out transition that marks the starting of total recoding time to the first sleep episode) for day- and night-time, averaged over five LD cycles.

### Immunohistochemistry

Adult heads were fixed and dissected in 4% formaldehyde in 1X PBS and then washed three times in PBS containing 0.2% Triton X-100 (PBST). Heads were then blocked in blocking solution (5% goat serum in 0.2% PBST). Primary antibodies were mouse-anti-PDF (Developmental Studies Hybridoma Bank), guinea pig-anti-Clockwork Orange (CWO) (gift from Dr. Paul Hardin laboratory), goat-anti-PER (gift from Dr. Michael Rosbash laboratory), rat-anti-TIM (gift from Dr. Amita Seghal laboratory); and secondary antibodies were Alexa Fluor 488, 568, and 647 (Invitrogen). Finally, wholemount dissected brains were washed again and mounted with Vectashield mounting media containing DAPI (Vector Laboratories). GFP-tagged dTau was directly visualized without anti-GFP antibody incubation.

### Confocal Microscopy

Visualization of *Drosophila* brain optical sections was performed on a Zeiss LSM800 confocal microscope. Image acquisition was made with 40X objective (oil-immersion) with optical zoom. For intensity quantification studies, laser parameters were maintained invariable.

### Sholl Analysis

Quantification of the PDF (Pigment Dispersing Factor) positive signal across LNv neuron projections was analyzed with Sholl analysis (Sholl, 1953), in which six concentric and evenly spaced (10 μm) rings are drawn. The centers of the rings are localized at the point where the first dorsal ramification starts in each hemisphere. The number of intersections per ring as well as the total number of intersections were compared using a one-way ANOVA statistical test. Scoring was performed blindly. FIJI software was used for axonal crosses, and quantification was according to software instructions.

### Statistics

Statistical analysis was performed using GraphPad Prism 8 (GraphPad Inc.). A Normality test was performed for every data set from the different experimental results and statistical tests were used accordingly. Details of statistical tests used in described in the figure legends. Statistical significance is indicated as ^∗^*p* < 0.05, ^∗∗^*p* < 0.01, ^∗∗∗^*p* < 0.001, and ^****^p < 0.0001 or NS if no statistical significance is obtained.

## Results

### Loss of Tau Alters Daily Rhythms and Sleep in *Drosophila melanogaster*

*Drosophila melanogaster* is a valuable model organism in investigating novel regulators (direct or indirect) of circadian rhythms and sleep. Locomotor activity in *Drosophila* is organized such that, in a 12:12 h light:dark (LD) cycle, flies exhibit peaks of activity during dawn and dusk (morning and evening peaks), with increases in activity typically occurring slightly before (anticipating) the lights-on and lights-off transitions (Figure 1). We found that tau deficient animals (dTau^–/–^) (Burnouf et al., 2016) display a different activity pattern than isogenic controls (w^1118^). Control animals showed expected day/night locomotor activities under a 12:12 h light:dark (LD) cycles (Figures 1A,C) with higher activity observed during the day than at night. Control flies also display a typical drop in activity (siesta) around mid-day between the two activity peaks, one at dawn and one at dusk (lights on: zeitgeber time (ZT) = 0, lights off: ZT = 12). Tau deficient flies were found to display higher total activity during the day (Figures 1B,D,E). We observed tau deficient flies to have significantly higher activity at mid-day, where control flies displayed a drop in their locomotor activity. In addition, tau deficient flies show a significantly higher peak of activity in the morning, compared to control flies (Figures 1A–E). However, we did not observe any change in activity patterns at night for the tau deficient flies (Figures 1A–E). In order to assess the robustness and integrity of the circadian clock, we monitored locomotor activity in the absence of any external stimulus (i.e., constant darkness, DD). Typically, in constant dark conditions, the morning activity peak in Drosophila shrinks and only the evening peak persists, reoccurring with a period of ∼23.8 h. Control and tau deficient flies were entrained for at least 3 days. Upon transition into DD, control and tau deficient flies maintained sustained differences in activity between subjective day and night (Figures 1A,B,F–H). As we previously observed in Light/Dark conditions, tau deficient flies showed significantly higher activity during the subjective day (sDay, Figure 1H). No changes in rhythmicity or in free-running period were observed in tau deficient flies (Table 1).

**FIGURE 2:**
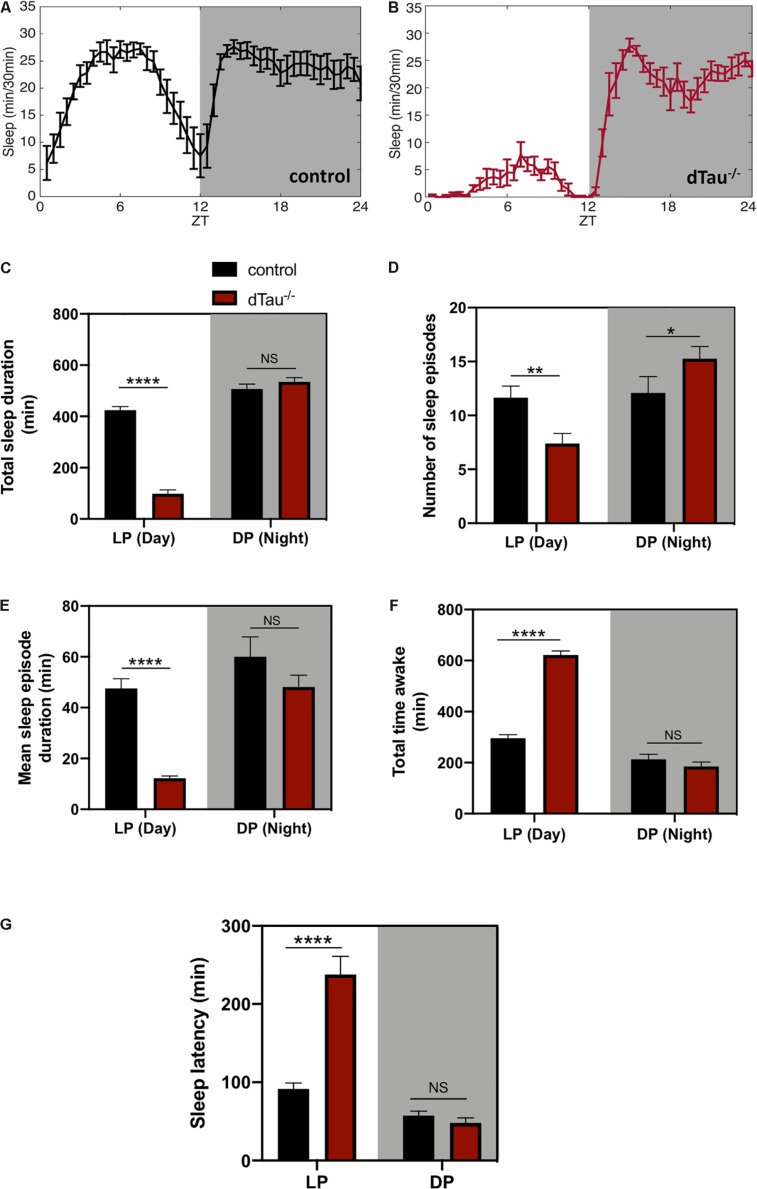
Loss of tau induces alterations in sleep in *Drosophila.* **(A,B)** Sleep plot representing the minutes of sleep per 30 min for control (in black) **(A)** and dTau mutant (in red) **(B)** of 7 day-old male flies in five averaged LD cycles. Gray shaded area represents the dark period (DP) or night. Results show a reduction in sleep in tau mutant flies in the day (LP) (white left side of the plots) but not in the night. **(C)** Total sleep duration quantification demonstrated significant differences between control and dTau^–/–^ flies. Total sleep quantification in LP and DP showed that the differences only occur during the day, and not during the night (DP). **(D)** Graph representing the number of sleep episodes in the LP and DP for dTau^–/–^ and control flies. Data show that the absence of tau significantly alters the number of sleep episodes, both in LP and DP. **(E)** Graph indicating differences in mean sleep episode duration, which shows a significant decrease in the case of dTau mutants specifically in LP and not in DP. **(F)** Total wake duration for light and dark periods. **(G)** Plot representing latency quantification. Data shows that latency to sleep was significantly increased during the day in Tau mutants, although there was no effect at night. Data represent mean and SEM, analyzed by non-parametric Mann–Whitney statistical test, with ^∗^*p* < 0.05, ^∗∗^*p* < 0.01, and ^****^p < 0.0001 or NS if no statistical significance (*n* = 20–25 flies).

In addition to eclosion and locomotor activity, circadian rhythms also drive other aspects of physiology and behavior, including sleep and metabolism. Circadian control of all of these processes relies not only on the intracellular clock, but also on networks of cells that interact to influence circadian outputs. In light of the results obtained, we went on to examine the sleep behaviors of these flies. Tau deficient flies showed sleep alterations when compared to controls (Figures 2A–E,G). Tau deficient flies sleep significantly less during the day, including the afternoon, with no significant changes observed during the night (Figure 2C). The number of sleep episodes are also significantly different at daytime and night time between controls and tau deficient flies (Figure 2D). When we measured the mean sleep episode duration of the dTau^–/–^ flies analysis demonstrated a significantly low- consolidated sleep during the daytime indicating a poor quality of sleep for the dTau^–/–^ flies (Figure 2E). Poor quality of sleep resulted in longer periods of time awake (Figure 2F). Importantly, the daytime sleep latency of tau deficient flies was surprisingly longer than that of control flies (Figure 2G). The increased time that it takes for the tau deficient flies to get to sleep is observed only during the day. These observed alterations in sleep are in accordance with similar alterations previously reported in mice (Cantero et al., 2010), supporting tau’s role in circadian modulation and sleep as a mechanism that is conserved among different species.

### Role of *Drosophila* Tau in the Core Circadian Neuronal Operation

**FIGURE 3:**
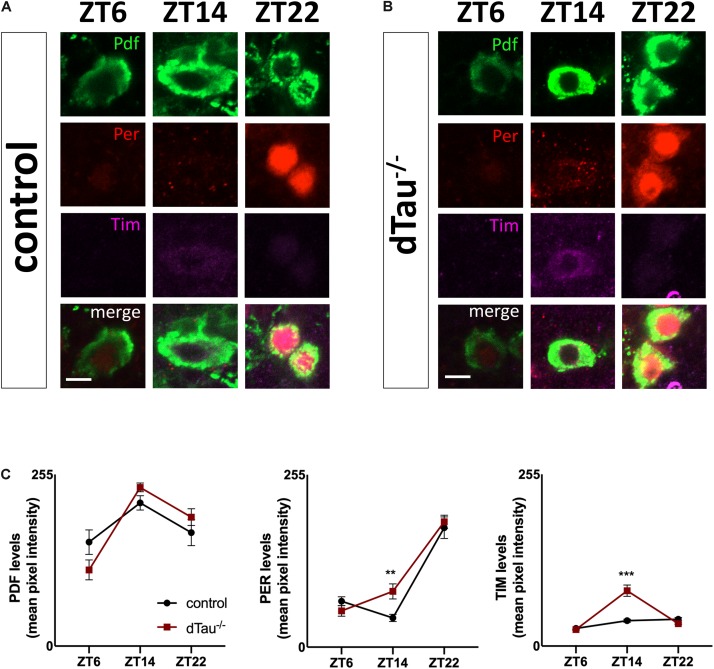
Minimal alteration of core clock operation in tau deficient flies. **(A,B)** Confocal images of LNv neurons immunostained for PDF, PER, and TIM, at different Zeitgeber times (ZT). Wholemount dissections of adult brains were done in control and dTau mutant of 7 day-old. **(C)** Quantification of PER, TIM, and PDF intensity levels across the indicated circadian time points (ZT-6, ZT-14, and ZT-22) shows significant increase in TIM and PER signals at ZT-14 when compared with controls. No changes were found at ZT-6 or ZT-22. Data represent mean and SEM analyzed by non-parametric Mann–Whitney statistical test (^∗∗^*p* < 0.01 and ^∗∗∗^*p* < 0.001; *n* = 12–14 hemispheres). Scale bar is 5 μm.

Next, we attempted to narrow down the cause of the locomotor and sleep defects in tau deficient flies. First, we tested whether rhythmic phenotypes observed in tau deficient flies might involve defective core clock operation. Two groups of central brain circadian neurons are particularly important for behavioral rhythms, including both morning (M) and evening (E) cells (Grima et al., 2004; Stoleru et al., 2004). The PDF-expressing small ventrolateral neurons (sLNvs or M cells) dictate morning activity as well as the rhythmicity in constant darkness (Renn et al., 1999; Blanchardon et al., 2001; Stoleru et al., 2004). Because of this latter feature, free running locomotor activity rhythms, the sLNvs are considered the major fly pacemaker neurons. The normal period of tau deficient flies implied that these clock cells functioned appropriately (Figure 1 and Table 1). To assess this more directly, we stained for periodic nuclear accumulation of PER and TIM in tau deficient flies and controls. Figure 3 shows a slightly significant difference in the magnitude of oscillatory dynamics between tau deficient flies and controls in sLNvs (Figure 3C). However, as the molecular clock is functional in tau deficient flies, this implies that tau protein regulates clock output function.

### *Drosophila* Tau Modulates Rhythmic Axonal Structural Remodeling

**FIGURE 4:**
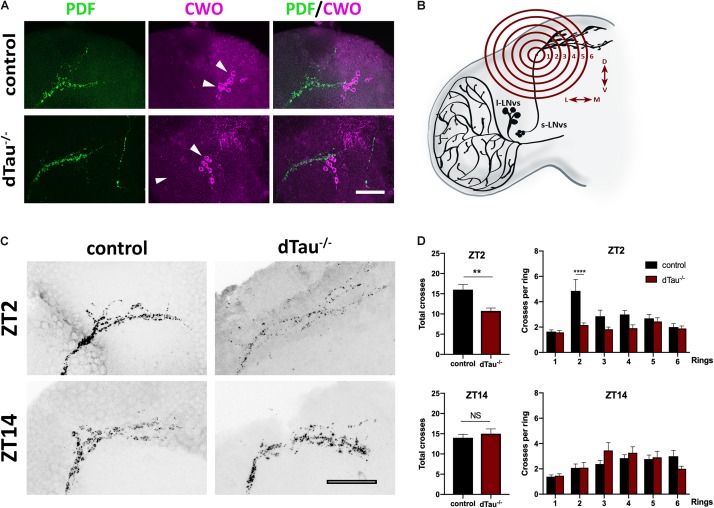
Effect of tau deficiency on daily rhythms of axonal structural remodeling. **(A)** Immunolabeling with anti-PDF and anti-CWO antibodies in LNv and DN neurons of adult brains from 7 day-old male flies. CWO immunostaining denotes the proper localization and morphology of DN neurons (arrow heads). Scale bar is 20 μm. **(B)** Scheme representing Sholl analysis in PDF-positive (black) LNv projections. Six concentric and evenly spaced (10 μm) rings (in red) are drawn. The centers of the rings are localized at the point where the first dorsal ramification starts in each hemisphere. **(C)** Representative confocal images of LNv neurons immunolabeled with anti-PDF antibody at ZT-2 and ZT-14, early morning and early evening, respectively. Panels show PDF-positive signal in LNv projections. Scale bar is 25 μm. **(D)** Plots representing Sholl analysis quantification at ZT2 and ZT14 for the corresponding genotypes. dTau^–/–^ flies showed a significant reduction in the spreading across the concentric rings specifically at ZT-2, when compared to controls. No changes were found at ZT-14. Data represent mean and SEM analyzed by one-way ANOVA test, with ^∗∗^*p* < 0.01 and ^****^p < 0.0001 or NS if no statistical significance (*n* = 12–14 hemispheres).

Given the effect of tau observed on daily activity and sleep behavior, we analyzed expression of PDF and CWO, which respectively label LNvs, including the sLNvs (or M cells), and all major circadian groups (including both M and E cells). These markers display normally in tau deficient brains and controls, and LNvs extended normal axonal and dendritic projections (Figure 4A). It has previously been shown that the dorsal projections of the sLNv neurons show rhythmic remodeling and that an operational clock is required for this circadian structural plasticity (Fernandez et al., 2008). In light of our previous results, we decided to assess if this process was affected in tau deficient flies. Day-night (ZT2 and ZT14) changes in PDF terminal morphology were measured in tau deficient flies and control flies (Figures 4C,D). The degree of axonal arborization was quantified by using an adaptation of Sholl’s method to study the dendritic branching pattern (Sholl, 1953; Fernandez et al., 2008). As illustrated in Figure 4B, the number of intersections between the concentric rings and the projections were determined. In controls, we observed, as expected, a significant difference in the axonal morphology between early morning (ZT2) and early night (ZT14). The decrease in the complexity of the axonal arbor of the PDF circuit at ZT14 is represented by fewer intersections. When we analyzed the effect of tau deficiency, we found a significantly reduced number of axonal crosses at ZT2, representing a reduction in the structural morphology of the sLNv (Figure 4D) that correlated with an increased circadian activity and decreased sleep at this given zeitgeber time (ZT2).

### Daily Expression of *Drosophila* Tau in PDF Neurons

**FIGURE 5:**
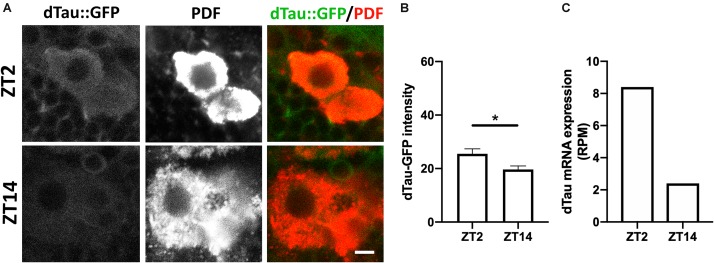
Modulation of tau protein levels during daily rhythms. **(A)** Representative images of immunolabeled 7 day-old male dTau-GFP fly brains with anti-PDF antibody at ZT-2 and ZT-14 (early morning and early night time-points). Scale bar is 10 μm. **(B)** Plot representing absolute intensity values of dTau-GFP signal in LNv somas at ZT-2 and ZT-14. Results demonstrate a significant difference between dTau fluorescent signal at the studied Zeitgeber times, with intensity values higher at ZT-2 and lower at ZT-14. **(C)** Plot representing dTau mRNA levels resulting from PDF neurons RNA-seq analysis (Abruzzi et al., 2017). Results demonstrate a reduction in dTau transcript levels at ZT-14 in PDF neurons when compared to dTau mRNA levels at ZT-2. Data represent mean and SEM analyzed by non-parametric Mann–Whitney statistical test (^∗^*p* < 0.05; *n* = 12–14 hemispheres).

To further investigate the role of tau in the structural plasticity of the PDF-positive sLNv neurons, we decided to analyze the expression pattern of dTAu in the sLNv neurons. To this aim, we took advantage of a *Drosophila* line expressing the endogenous dTau tagged with GFP. Interestingly, we found that dTau expression in PDF-positive neurons changes depending on the time of day (Figure 5). Quantification of dTau protein immunostaining levels revealed that dTau expression was significantly higher in early morning than in early night (Figure 5B). It is important to mention that dTau and PDF expression is increased in the morning, paralleling this peak of activity. In addition, we analyzed dTau gene expression using available RNA-seq data sets from LNv (PDF positive) neurons (Abruzzi et al., 2017). Strikingly, a 6-fold increase in dTau gene expression was found in the early morning (ZT2) (Figure 5C), suggesting tau expression is necessary for the observed structural plasticity of the sLNv terminals.

## Discussion

Our results show major abnormalities in circadian activity and sleep in dTau^–/–^ flies, including higher morning activity and reduced sleep during the day-light period of the bimodal circadian rhythm (Figures 1–2). These results are also in agreement with previous results obtained in tau KO mice (Cantero et al., 2010). Sleep alterations are observed as a function of normal aging, yet in AD, an exacerbated dysregulation of circadian sleep patterns increases in severity as the disease progresses, contributing to impairment in cognitive function (Musiek et al., 2015; Homolak et al., 2018).

Here, similar patterns of sleep to those observed in human patients with AD and related tauopathies (Aldrich et al., 1989; Logsdon et al., 1998; Vitiello and Borson, 2001) were observed in dTau^–/–^ flies (Figure 2). The prolonged sleep latency observed in the dTau^–/–^ flies following lights-off seems to mimic the sundowning syndrome observed in human tauopathic patients (Aldrich et al., 1989) to some extent, supporting tau loss-of-function as a possible pathogenic mechanism of the disease.

Experimental evidence suggests that slow and fast neural oscillations are strongly involved in motor behavior and sleep (Saleh et al., 2010; Posluszny, 2014; Niethard et al., 2018). In this regard, previous results in tau KO mice have indicated slowing of theta rhythms (5–11 Hz) in the hippocampus, and loss of functional connectivity between the hippocampus and the frontal cortex in the gamma range (30–80 Hz) (Cantero et al., 2011). Our results in dTau^–/–^ flies agree with previous observations in tau KO mice on motor behavior and sleep, however, future studies showing neural activity recordings of clock neurons of dTau^–/–^ flies may uncover alterations on electrophysiological properties and will further confirm the role tau protein appears to play in the regulation of these neuronal responses and behaviors. In addition, a recent study has shown that abnormal hyperphosphorylated tau alters circadian clock operation supporting tau loss-of-function (Stevanovic et al., 2017) similar to our obtained results in dTau^–/–^ flies.

The circadian alterations observed in tau deficient flies may involve aberrant environmental perception, defective core clock operation, or perhaps altered clock output. We first considered whether tau deficiency affected visual phototransduction. This was conceivable given the altered light-on responses (Figure 1). However, electrophysiological recordings performed in the dTau^–/–^ line did not show any abnormalities (Burnouf et al., 2016). In addition, electroretinogram recordings in young tau KO mice are normal (Rodriguez et al., 2018), suggesting loss of tau does not induce developmental defects on retinal neurons.

During normal clock operation in *Drosophila*, levels of per and tim mRNA rise during the day. The two proteins start to accumulate, initially in the cytoplasm, and then around the middle of the night in the nucleus (Zheng and Sehgal, 2012). TIM stabilizes PER in the cytoplasm, and is required to transport it to the nucleus. Nuclear localization of the two proteins is also regulated by specific importins (Jang et al., 2015) and appears to be temporally regulated (Curtin et al., 1995; Meyer et al., 2006). Nuclear localization of PER and TIM coincides with the decline of their mRNA levels due to negative autoregulatory feedback by the proteins. PER and TIM cannot bind DNA, but regulate transcription by inhibiting their transcriptional activators, Clock and Cycle. We observed minimal but significant alterations in the core clock proteins in tau deficient flies (Figure 3). Alterations in tau proteostasis leading to detrimental loss-of-function effects have been shown in Alzheimer’s disease and related tauopathies where pathogenic tau alters nucleocytoplasmic transport by interacting with components of the nuclear pore complex (Eftekharzadeh et al., 2018). Future studies would be necessary to rule out the role tau protein plays in modulating nucleocytoplasmic transport in clock neurons.

In *Drosophila*, rhythmic locomotor cycles rely on the activity of approximately 150 neurons grouped in seven clusters (Helfrich-Forster, 2003; Shafer et al., 2006; Dubowy and Sehgal, 2017). Work from many laboratories have pointed to the sLNvs as critical for circadian control of locomotor rhythmicity (Renn et al., 1999; Blanchardon et al., 2001; Grima et al., 2004; Stoleru et al., 2004, 2005). The sLNv neurons undergo circadian remodeling of their dorsal axonal projections allowing these circadian pacemaker neurons to change synaptic contacts across the day (Fernandez et al., 2008; Gorostiza et al., 2014). This plasticity is critical given the role sLNv neurons play in the Drosophila circuit connecting circadian clock neurons to sleep promoting neurons (Guo et al., 2018). Our results show that loss of tau impinges on circadian structural plasticity and temporal dynamics of the sLNv axonal projections (Figure 4). A similar effect of tau protein on structural plasticity has also been recently described in hippocampal granule neurons (Bolos et al., 2017). Structural alterations on terminal projections of sLNv could be promoted depending on tau protein’s ability to interact with microtubules. In this regard, future studies are needed to further explore the role disease-associated hyperphosphorylated tau plays in structural plasticity and pathophysiology of clock neurons in Alzheimer’s disease and related tauopathy. Additionally, disease-associated tau could play a crucial role in circadian disruption and sleep alterations observed in AD patients through interaction with the actin cytoskeleton in the terminal projections, altering circadian neuronal plasticity (Petsakou et al., 2015).

On the other hand, dysregulation of tau proteostasis could have implications beyond mere structural changes in terminal projections. Tau, through the regulation of cytoskeleton dynamics, could be modulating the biology of melatonin receptors, which play important roles in the sleep-wake cycle (Jarzynka et al., 2006, 2009).

We have found that dTau levels change during the day, presumably fulfilling the cytoskeletal demands during periods of structural remodeling, like the one observed for the dorsal projections of the sLNv neurons. However, it is unknown how tau levels are regulated in this context. Evidence is emerging for post-transcriptional clock regulators (Lim and Allada, 2013). As key regulators of mRNA stability and translation, microRNAs (miRNAs) are implicated in multiple aspects of time keeping. miRNAs are capable of binding to and silencing many target transcripts, providing an additional level of regulation that complements canonical transcriptional pathways. miR-219 and miR-132 were early miRNAs identified to regulate mammalian clocks (Cheng et al., 2007). Recently, it has been described that miR-219 dysregulation promotes neurodegeneration through posttranscriptional regulation of tau. Since miR-219 is the only conserved miRNA able to modulate tau across multiple species, it would be important, in future studies, to explore its mechanisms of regulating tau in clock neurons.

## Conclusion

In conclusion, our results support the role tau protein plays in the regulation of circadian activity and sleep in *Drosophila*, presumably through the modulation of structural and temporal dynamics of the cytoskeleton in terminal projections of the circadian pacemaker neurons. This conclusion is based on the fact that tau deficiency disrupts the temporal dynamics of circadian pacemaker neurons. Our findings are also consistent with previously described observations on the possible role of tau on sleep regulation in mice. Therefore, further validation in mouse models and accompanying behavioral studies would be valuable. Further studies will provide a better understanding of the functions of tau and advance our understanding of the role it plays in circadian biology and sleep behavior.

## Data Availability Statement

All datasets generated for this study are included in the article.

## Author Contributions

MA, MEA, JC, and IS-M conceived and co-ordinated the study. IS-M and MA designed the experiments. MA, MEA, GL, and CK conducted the experiments and analyzed the data. IS-M wrote the first draft of the manuscript. CK and JJ contributed to the materials and critically revised the initial manuscript draft. All the authors discussed and commented on the final manuscript.

## Conflict of Interest

The authors declare that the research was conducted in the absence of any commercial or financial relationships that could be construed as a potential conflict of interest.
